# Endometriosis and Adverse Pregnancy Outcomes: A Nationwide Population-Based Study

**DOI:** 10.3390/jcm12165392

**Published:** 2023-08-19

**Authors:** Hee Jeung Lim, Jiyu Sun, Banhyang Min, Myungeun Song, Tae Hun Kim, Byoung Jae Kim, Kyu Ri Hwang, Taek Sang Lee, Hye Won Jeon, Sun Min Kim

**Affiliations:** 1 Department of Obstetrics and Gynecology, Seoul National University College of Medicine, Seoul 03080, Republic of Korea; heejeunglim@gmail.com (H.J.L.); moonlit0@naver.com (B.M.); christy2875@naver.com (M.S.); coolluck1979@gmail.com (T.H.K.); bjkimog@nate.com (B.J.K.); orangemd0126@hanmail.net (K.R.H.); tslee70@gmail.com (T.S.L.); jshmom04@naver.com (H.W.J.); 2 Department of Obstetrics and Gynecology, Seoul Metropolitan Government Seoul National University Boramae Medical Center, Seoul 07061, Republic of Korea; 3 Integrated Biostatistics Branch, Division of Cancer Data Science, National Cancer Center, Goyang-si 10408, Republic of Korea; jiyu.sun0@gmail.com

**Keywords:** endometriosis, pregnancy outcome, preeclampsia, placenta previa, postpartum hemorrhage, stillbirth, placental abruption, cesarean delivery

## Abstract

Endometriosis is a major cause of infertility, and considering its pathophysiology, it is expected to affect pregnancy outcomes as well. This study aimed to evaluate whether endometriosis is associated with adverse pregnancy outcomes after successful conception. Data from singleton pregnancy deliveries between January 2014 and October 2019 were obtained from the Korean Health Insurance Review and Assessment Service database. We compared the clinical characteristics and adverse pregnancy outcomes of women with and without endometriosis. A total of 1,251,597 pregnant women were enrolled; of these, 32,951 (2.6%) were assigned to the endometriosis group. Women with endometriosis had significantly more adverse pregnancy outcomes than those without endometriosis. Adverse pregnancy outcomes associated with endometriosis included preterm labor, preterm birth, preeclampsia, fetal growth restriction, placenta previa, placental abruption, antepartum and postpartum hemorrhage, and stillbirth. This study also showed an increased risk of postpartum hemorrhage, blood transfusion, uterine artery embolization, and cesarean hysterectomy in the endometriosis group compared to the non- endometriosis group. The cesarean delivery rate was significantly higher in the endometriosis group than in the non-endometriosis group, even after excluding cases of antenatal obstetric complications that could increase the risk of cesarean delivery. Women with endometriosis not only have difficulty conceiving, but also have a significantly higher risk of adverse pregnancy outcomes.

## 1. Introduction

Endometriosis is an estrogen-dependent chronic disease and is defined as the presence of endometrial tissue outside the uterus; it can involve the ovaries, fallopian tubes, and posterior cul-de-sac [1]. It significantly contributes to pelvic pain and infertility in women. Although the prevalence of endometriosis is reported to range from 6% to 10% in the general female population, it is one of the most representative causes of infertility, with a prevalence of approximately 50% in women with infertility [2,3]. Considering its pathophysiology, endometriosis is expected to have negative effects on pregnancy outcomes. With pregnancy, hormonal and inflammatory changes occur that are regulated to ensure proper decidualization and placentation as well as to maintain pregnancy and active labor at term. Similarly, there is a shift in hormones and inflammatory factors in endometriosis that could overlap and ultimately interfere with the processes involved in pregnancy. Cytokines, proteases, and matrix metalloproteinases play a role in proper decidualization, which is important for successful blastocyst implantation. Changes in the immune cell population observed in endometriosis may disrupt the inflammatory pathways regulated by decidua cells to affect proper trophoblast invasion and implantation [4]. Proinflammatory cytokines interleukin (IL)-1 and IL-6 have been shown to increase in endometriosis, and are known to be associated with myometrial contractility in active labor and preterm birth [5,6]. In addition to inflammatory effects, endometriosis is known to infiltrate pelvic tissues and cause adhesions or anatomical distortions over time, which could affect pregnancy outcomes. The endometrial tissue can invade the myometrium, leading to defective deep placentation or improper remodeling of myometrial spiral arteries that could lead to placental complications or hypertensive disorders in pregnancy [7,8]. Pelvic adhesions caused by endometriosis could make vaginal delivery or cesarean section difficult.

Previously, some studies on endometriosis and adverse pregnancy outcomes have reported a statistically significantly elevated risk of spontaneous abortion, preterm birth, cesarean delivery, and placental complications in women with endometriosis [1,9,10,11]. In a Danish cohort study of 82,793 women, patients with endometriosis had two times the risk of cesarean delivery, and about a 73% greater risk of preterm birth [9]. Another registry-based study of 91,825 women that included not only endometriosis, but also uterine fibroid patients, showed the greatest risk of placental abnormalities among other pregnancy complications, with a risk increased by about 65% in the endometriosis group compared to the non-endometriosis group [10]. Some studies have questioned whether endometriosis is truly a high-risk group for pregnancy complications [12,13,14]. A retrospective study of 108 women conducted in Japan did not observe significant differences in adverse pregnancy outcomes between the endometriosis and non-endometriosis patients, including cesarean delivery and preterm birth, therefore questioning the association between endometriosis and pregnancy outcomes [12]. Additionally, a matched case–control study of 274 women showed no greater risk of placental complications in patients with endometriosis [13]. However, these studies that have reported no relationship between endometriosis and pregnancy outcomes had the limitation of small sample sizes. Regarding whether endometriosis increases the risk of hypertensive disorders, gestational diabetes, and postpartum hemorrhage, past studies’ findings have been less conclusive, and this topic warrants further studies.

This study aimed to compare the adverse pregnancy outcomes between women with and without endometriosis based on the nationwide claims database of the Korean Health Insurance Review and Assessment Service (HIRA). 

## 2. Materials and Methods

This retrospective cohort study was conducted using large-scale data from the Korean HIRA database, a South Korean government-operated database that stores information on almost all healthcare use of the South Korean population (>50 million people) [15]. The national health insurance reviews data such as clinical information, diagnoses, and comorbidities in relation to hospital medical procedures and drug prescriptions for appropriate financial compensation. Such information, stored by the Korean HIRA database, has proven to be an important resource for research and has been evaluated to have high validity and reliability [16,17].

We collected data on women who gave birth in South Korea between 1 January 2010 and 31 December 2019 from the HIRA database. Information ranging from diagnosis of endometriosis to pregnancy outcomes was searched based on the International Classification of Diseases, 10th Revision (ICD-10) codes (World Health Organization, Geneva, Switzerland). As the estimated due date is calculated by adding 266 days to the date of conception, the index date was defined as 266 days before delivery, and considering that the puerperium period lasts six weeks after giving birth, the follow-up end date was defined as 42 days after delivery. The follow-up duration covered the entire pregnancy and puerperal period. 

**Figure 1:**
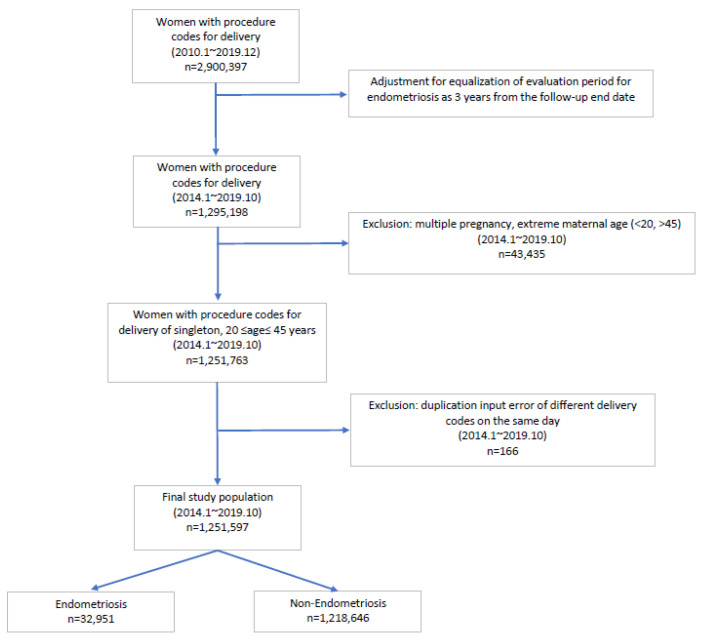
Enrollment flowchart of the study population. The flowchart shows the inclusion and exclusion criteria and the process of final selection of participants for the study.

The patients were divided into endometriosis and non-endometriosis groups, as shown in Figure 1. The evaluation period of whether the participant was diagnosed with endometriosis was arbitrarily set to three years from the follow-up end date to ensure that every participant had equal time to be exposed to endometriosis. Participants who had multiple pregnancies and those with advanced maternal age were excluded, as multiple pregnancies and both extremes of maternal age can increase pregnancy complications. As a result, patients aged 20–45 years, and with only singleton pregnancies, were included. The cases with input error, such as different delivery codes on the same day for a patient, were excluded. In addition, the outcomes of only the first deliveries of women who had given birth multiple times during the study period were included to prevent repeated analyses of the same patient. Patients who were diagnosed with endometriosis were identified using the ICD-10 code for endometriosis (N80). Because this study used health insurance claim data rather than reviewing medical records, information on how endometriosis was diagnosed, or on the severity and staging of endometriosis, could not obtained due to the nature of big data.

Patient characteristics that were investigated included maternal age at delivery, parity, underlying medical conditions such as pre-pregnancy hypertension and pre-pregnancy diabetes mellitus, and use of assisted reproductive technology. Other demographics such as the patient’s height, weight, and body mass index were not provided by the database. Parity information of the participant was deduced from the delivery-related behavior code, and the study population was classified based on parity. Assisted reproductive technology was identified when diagnostic codes for intrauterine insemination and in vitro fertilization were assigned.

Pregnancy complications known to be associated with maternal or fetal morbidity and mortality were considered to represent adverse pregnancy outcomes in this study, and those that had corresponding diagnostic codes available in the database were finally selected. In this study, adverse pregnancy outcomes included the following pregnancy complications: preterm labor (O60.0); preterm birth (O60.1, O60.3); pregnancy-associated hypertension (O11, O13, O14, O15); fetal growth restriction (O36.5); gestational diabetes mellitus (O24.4); placenta previa (O44); placental abruption (O45.9); dystocia (O61–O66); labor and delivery complicated by fetal distress (O68); fetal malpresentation (O32); antepartum hemorrhage (O46); postpartum hemorrhage (O72); and stillbirth (O36.4). Gestational hypertensive disorders included diagnostic codes for gestational hypertension (O13), preeclampsia (O14), superimposed preeclampsia (O11), and eclampsia (O15). We analyzed the incidence of postpartum hemorrhage and additional interventions for postpartum hemorrhage, such as blood transfusion, uterine artery embolization, and cesarean hysterectomy, using the appropriate codes. The incidence of cesarean delivery and operative vaginal delivery with extraction using vacuum or forceps was also extrapolated from the database to compare the rates of cesarean delivery and operative vaginal delivery between the groups. Because antepartum obstetric complications can increase the risk of cesarean delivery, we attempted to analyze the relationship between the cesarean delivery risk and endometriosis after excluding the cases with antepartum obstetric complications such as preterm birth, gestational hypertensive disorders, fetal growth restriction, gestational diabetes mellitus, placenta previa, and placental abruption.

The baseline characteristics are presented as frequency (percentage) and as a mean (standard deviation) for categorical and continuous variables, respectively. The characteristics of patients in the endometriosis and non-endometriosis groups were compared using the chi-square test or two-sample t-test, depending on the type of variable. Moreover, differences in pregnancy outcomes between the two groups were assessed using the chi-squared test. Poisson regression with robust error variance was used to calculate the relative risks (RR) and 95% confidence intervals (CI) to estimate the relationship between endometriosis and pregnancy complications. The models were adjusted for maternal age, parity, use of assisted reproductive technology, pre-pregnancy diabetes mellitus, and pre-pregnancy hypertension. Two-sided *p* < 0.05 was considered statistically significant. Statistical analyses were performed using SAS software (version 9.4; SAS Institute, Cary, NC, USA).

The Institutional Review Board of the Seoul Metropolitan Government-Seoul National University Boramae Medical Center approved the exemption from review (IRB no. 70-2020-188), since this study used big data that did not include personal identifiable information.

## 3. Results

In this study, a total of 1,251,597 women with singleton deliveries were enrolled; 32,951 (2.6%) were reported to have endometriosis and 1,218,646 (97.4%) were not diagnosed with endometriosis (Figure 1). The average maternal age at delivery, as well as the rates of nulliparity and assisted reproductive technology-induced pregnancies, were higher in the endometriosis group than in the non-endometriosis group. No significant difference was noted in the presence of underlying diabetes mellitus between the groups; however, the pre-pregnancy hypertension rate was higher in the endometriosis group than in the non-endometriosis group (Table 1).

Most adverse pregnancy outcomes occurred significantly more often in the endometriosis group than in the non-endometriosis group (Table 2). The RR was increased in the endometriosis group for major adverse outcomes such as preterm labor, preterm birth, preeclampsia, fetal growth restriction, placenta previa, placental abruption, labor and delivery complicated by fetal distress, fetal malpresentation, cesarean delivery, antepartum and postpartum hemorrhage, stillbirth, postpartum hemorrhage, blood transfusion, and cesarean hysterectomy (Table 3). Even after adjusting for confounding factors such as maternal age, parity, use of assisted reproductive technology, pre-pregnancy hypertension, and diabetes mellitus, the RR for pregnancy complications was higher in the endometriosis group than in the non-endometriosis group.

The relationship between cesarean delivery rate and endometriosis was analyzed further only in cases without antepartum obstetric complications that could increase the risk of cesarean delivery. The cesarean delivery rate was significantly higher in the endometriosis group than in the non-endometriosis group in this subgroup analysis after adjusting for maternal age, parity, assisted reproductive technology, and previous hypertension and diabetes mellitus (Table 4).

## 4. Discussion

In this study, women with endometriosis demonstrated an increased risk of adverse pregnancy outcomes compared with those without endometriosis. The endometriosis group had higher RRs of preterm labor, preterm birth, preeclampsia, fetal growth restriction, placenta previa, placental abruption, labor and delivery complicated by fetal distress, fetal malpresentation, cesarean delivery, antepartum and postpartum hemorrhage, and stillbirth.

This increased risk of preterm labor, preterm birth, and pregnancy loss is consistent with the findings of previous studies [9,11]. This may be related to the main pathogenesis of endometriosis causing a chronic inflammatory environment with increased levels of proinflammatory cytokines such as IL-1β, IL-6, and tumor necrosis factor-α [18]. Such inflammation is associated with inducing uterine cervix remodeling and uterine contraction, and is known to be a major cause of spontaneous preterm birth [19,20].

Chronic inflammation due to endometriosis may affect decidualization, which could interfere with proper trophoblast invasion. Decidual cells release cytokines and growth factors that help to regulate processes of appropriate trophoblast invasion, which could be interrupted by changes caused by endometriosis, particularly in IL-11 levels [4]. This could explain the constant finding of a greater risk of placental anomalies in women with endometriosis in previous studies and in this study.

The association between postpartum hemorrhage and endometriosis has been inconsistent across studies [9,10,21]. In the present study, the rate of postpartum hemorrhage, including rates of blood transfusion, uterine artery embolization, and cesarean hysterectomy, was significantly higher in women with endometriosis than in those without endometriosis. When women with endometriosis become pregnant, ectopic endometrial implants also undergo histological changes of decidualization and are transformed into well-vascularized tissue as the pregnancy progresses [21]. These tissues can be prone to unpredictable dysfunctional bleeding because endometriosis is progesterone-resistant, and focal necrosis of the ectopic endometrium surrounding the distended veins could occur during pregnancy [22,23].

In this study, we found an increased risk of gestational hypertensive disorders in women with endometriosis, with an adjusted RR of 1.208 (1.130–1.292). The association between endometriosis and hypertension has been inconsistent in many studies. A systematic review that included over a million women showed no associated risk of hypertensive disorders with endometriosis [1]. However, the limitation of inconsistently adjusted confounding factors applied among the multiple sets of data was noted in the systemic review. Conversely, a retrospective study involving 91,825 women, conducted using an all-payer claims database showed an increased risk of pregnancy-induced hypertension [11]. A prospective cohort study of only laparoscopically confirmed endometriosis patients reported a 30% greater risk of hypertensive disorders in endometriosis patients than in non-endometriosis patients [24]. This study included one of the largest study populations, and the results support the association of endometriosis with an increased risk of gestational hypertensive disorders. The mechanism that could explain this observation could include improper trophoblast invasion due to changes in cytokines and thicker junctional zones of the myometrium in women with endometriosis [4,25]. As transformation of the spiral arteries in the junctional zone of the myometrium is an essential process for normal placenta formation, different features of the junctional zones of endometriosis patients could induce abnormal placental function and thus increase the risk of gestational hypertensive disorders [22,25,26].

The endometriosis group did not show a significantly increased risk of gestational diabetes mellitus compared to the non-endometriosis group after adjusting for confounding factors in this study. A recent meta-analysis study observed an increased risk of gestational diabetes mellitus in endometriosis patients with an OR of 1.23 (1.07–1.51), and showed that the risk tripled in stage III-IV endometriosis compared to stage I-II endometriosis [27]. The prospective study on laparoscopically confirmed endometriosis also demonstrated a greater risk of gestational diabetes mellitus in endometriosis patients [24]. Although these studies showed associations between GDM and endometriosis, the mechanism behind this association is still unclear. Gestational diabetes mellitus seems to increase in assisted-reproductive-technology-induced pregnant women, which could be explained by how many of the infertility patients have polycystic ovarian syndrome, a condition that increases the risk of diabetes. Yet, the recent meta-analysis study has shown that the risk of gestational diabetes mellitus does not specifically increase in patients who have used assisted reproductive technology among those with endometriosis [27]. As of now, it seems that there is a need to further investigate whether gestational diabetes mellitus is associated with endometriosis.

The risk of cesarean delivery was still significantly higher in the endometriosis group after adjusting for confounding factors or antepartum obstetric complications which would increase the risk of cesarean delivery. The increased risk can be attributed to the potential impact of endometriosis on pelvic adhesions and the distortion of pelvic structures [2], both of which have the potential to disrupt the natural progression of labor and amplify the likelihood of requiring a cesarean delivery. Furthermore, it is worth noting that women afflicted with endometriosis might lean towards opting for cesarean deliveries. This inclination could be attributed to their desire to circumvent the uncertainties associated with labor, instead preferring the foreseeable risks tied to an elective cesarean delivery—especially in cases in which they have previously grappled with chronic pelvic pain or encountered challenges when conceiving [10].

One of the strengths of this study is the evaluation of a large-scale study population using the national database with a total of 1,251,597 patients, making it one of the largest cohort studies investigating the association between endometriosis and adverse pregnancy outcomes. The prevalence of endometriosis in this population was 2.6%, which was similar to the prevalence reported in two other studies on pregnant women with endometriosis [9,21].

Our study was able to avoid the error of allowing the more recently delivered study participants to have a higher chance of being diagnosed with endometriosis within the study period by setting a timeframe of three years for each participant’s diagnosis of endometriosis.

However, it is important to acknowledge that research conducted using big data has inherent limitations compared with studies based on individual patients’ medical records, such as a lack of specificity. There was a lack of information on how endometriosis was diagnosed, and we were unable to determine whether endometriosis was clinically diagnosed or surgically confirmed. Although a study with only surgically confirmed endometriosis patients would be ideal, accumulating as much data as possible using the nationwide database would be difficult and time-consuming. Information on whether the patient has been treated for endometriosis may provide insight into whether treating endometriosis before pregnancy leads to an improved pregnancy outcome. Data on the duration of having an endometriosis diagnosis were also unavailable; this information may have been used to analyze whether chronic endometriosis leads to a greater risk of pregnancy complications. The severity of the patient’s endometriosis was not provided by this database, although it could have been useful for further understanding the association between endometriosis and adverse pregnancy outcomes. Besides the details of the diagnosis of endometriosis, information regarding body mass index, a useful demographic, could not be obtained from this database. There could have been individual bias when submitting diagnostic codes into the database, with different diagnostic codes being entered for the same medical situation depending on the hospital or physician in charge. National data, however, even with their limitations, have the advantage of being large-scale. South Korea has a well-established medical insurance system managed by the government; therefore, the data submitted into the Korean HIRA Service database reflect actual medical care and have a high degree of reliability.

Despite our analysis of extensive nationwide big data, it is important to note that the study population remained ethnically homogenous, primarily comprising individuals of South Korean descent. Subsequent research endeavors should aim to assess the consistency of findings across various ethnic groups. In addition, future studies could investigate whether the risk of adverse pregnancy outcomes increases with the severity of endometriosis, or if treating endometriosis before pregnancy improves pregnancy outcomes.

## 5. Conclusions

In this study, which was performed using the national database, endometriosis was found to be significantly associated with adverse pregnancy outcomes, such as preterm labor, preterm birth, gestational hypertensive disorders, fetal growth restriction, placenta previa, placenta abruption, antepartum and postpartum hemorrhage, stillbirth, and cesarean delivery. Moreover, postpartum blood transfusion, uterine artery embolization, and cesarean hysterectomy were performed more frequently in the endometriosis group than in the non-endometriosis group. These findings emphasize the need to consider pregnancy in women with a history of endometriosis as high-risk, requiring special antepartum, intrapartum, and postpartum management.

## Figures and Tables

**Table 1:** Clinical characteristics of the study population.

Characteristics	Endometriosis Group (n = 32,951)	Non-Endometriosis Group (n = 1,218,646)	*p* Value
Maternal age, mean ± SD (in years)	33.1 ± 4.0	31.3 ± 4.3	<0.001
Parity			<0.001
0	30,630 (93.0%)	1,093,695 (89.8%)	
≥1	2321 (7.0%)	124,951 (10.2%)	
Assisted reproductive technology			<0.001
No Yes	30,837 (93.6%) 2114 (6.4%)	1,195,748 (98.1%) 22,898 (1.9%)	
Pre-pregnancy diabetes mellitus			0.810
No	32,910 (99.9%)	1,217,071 (99.9%)	
Yes	41 (0.1%)	1575 (0.1%)	
Pre-pregnancy hypertension			0.037
No	32,940 (99.97%)	1,218,430 (99.98%)	
Yes	11 (0.03%)	216 (0.02%)	

SD, standard deviation.

**Table 2:** Comparisons of adverse pregnancy outcomes between the two groups in the study population.

Characteristics	Endometriosis (n = 32,951)	Non-Endometriosis (n = 1,218,646)	*p* Value
Preterm labor	7794 (23.7%)	205,994 (16.9%)	<0.001
Preterm birth	773 (2.4%)	14,924 (1.2%)	<0.001
Gestational hypertensive disorders including preeclampsia	1290 (3.9%)	34,044 (2.8%)	<0.001
Fetal growth restriction	1110 (3.4%)	25,584 (2.1%)	<0.001
Gestational diabetes mellitus	6674 (20.3%)	228,118 (18.7%)	<0.001
Placenta previa	1552 (4.7%)	19,767 (1.6%)	<0.001
Placental abruption	112 (0.3%)	1756 (0.1%)	<0.001
Cesarean delivery	18,108 (55.0%)	552,451 (45.3%)	<0.001
Vacuum or forceps extraction delivery	2061 (6.3%)	92,352 (7.6%)	<0.001
Dystocia	2477 (7.5%)	108,864 (8.9%)	<0.001
Labor and delivery complicated by fetal distress	1632 (5.0%)	52,810 (4.3%)	<0.001
Malpresentation of fetus	2539 (7.7%)	61,833 (5.1%)	<0.001
Antepartum hemorrhage	617 (1.9%)	18,049 (1.5%)	<0.001
Postpartum hemorrhage	2587 (7.9%)	92,006 (7.6%)	0.041
Stillbirth	120 (0.4%)	2257 (0.2%)	<0.001
Uterine artery embolization	161 (0.5%)	2432 (0.2%)	<0.001
Blood transfusion	403 (1.2%)	6293 (0.5%)	<0.001
Cesarean hysterectomy	27 (0.08%)	306 (0.03%)	<0.001

**Table 3:** Association of endometriosis with adverse pregnancy outcomes after adjusting for confounding factors.

Characteristics	Crude Relative Risk	95% CI of RR		*p* Value	Adjusted * Relative Risk	95% CI of RR		*p* Value
		Lower	Upper			Lower	Upper	
Preterm labor	1.399	1.372	1.427	<0.001	1.360	1.334	1.388	<0.001
Preterm birth	1.916	1.784	2.058	<0.001	1.854	1.726	1.992	<0.001
Gestational hypertensive disorders	1.371	1.283	1.466	<0.001	1.208	1.13	1.292	<0.001
Fetal growth restriction	1.605	1.513	1.702	<0.001	1.493	1.407	1.584	<0.001
Gestational diabetes mellitus	1.082	1.059	1.106	<0.001	0.989	0.968	1.011	0.307
Placenta previa	2.904	2.761	3.054	<0.001	2.145	1.769	2.601	<0.001
Placental abruption	2.359	1.950	2.855	<0.001	2.145	1.769	2.601	<0.001
Cesarean delivery	1.212	1.200	1.224	<0.001	1.103	1.092	1.114	<0.001
Vacuum or forceps extraction delivery	0.825	0.791	0.861	<0.001	0.864	0.828	0.902	<0.001
Dystocia	0.842	0.810	0.874	<0.001	0.884	0.851	0.919	<0.001
Labor and delivery complicated by fetal distress	1.143	1.089	1.199	<0.001	1.148	1.094	1.204	<0.001
Malpresentation of fetus	1.519	1.462	1.578	<0.001	1.370	1.318	1.423	<0.001
Antepartum hemorrhage	1.264	1.168	1.369	<0.001	1.161	1.071	1.258	0.000
Postpartum hemorrhage	1.040	1.002	1.080	0.041	1.091	1.051	1.133	<0.001
Stillbirth	1.966	1.637	2.362	<0.001	1.902	1.582	2.287	<0.001
Uterine artery embolization	2.448	2.088	2.871	<0.001	2.074	1.765	2.436	<0.001
Blood transfusion	2.368	2.143	2.618	<0.001	2.071	1.872	2.291	<0.001
Cesarean hysterectomy	3.263	2.202	4.836	<0.001	4.042	2.715	6.018	<0.001

CI, confidence intervals; RR, relative risk. * Adjusted for maternal age, parity, assisted reproductive technology, pre-pregnancy diabetes, and pre-pregnancy hypertension.

**Table 4:** Comparison of cesarean delivery rates only in cases without antepartum obstetric complications.

Outcome	Total (N = 594,319)		Endometriosis Group (N = 13,432)		Non-Endometriosis Group (N = 580,887)		Crude Relative Ratio	95% CI of RR		*p* Value	Adjusted * Relative Ratio	95% CI of RR		*p* Value
	N	%	N	%	N	%		Lower	Upper			Lower	Upper	
Cesarean delivery	297,120	50.0	7796	58.0	289,324	49.8	1.165	1.148	1.183	<0.001	1.066	1.050	1.081	<0.001

* Adjusted for maternal age, parity, assisted reproductive technology, pre-pregnancy diabetes, and pre-pregnancy hypertension.

## Data Availability

Data are contained within the article.
